# Treatment with the PPARγ Agonist Pioglitazone in the Early Post-ischemia Phase Inhibits Pro-inflammatory Responses and Promotes Neurogenesis Via the Activation of Innate- and Bone Marrow-Derived Stem Cells in Rats

**DOI:** 10.1007/s12975-017-0577-8

**Published:** 2017-11-06

**Authors:** Tomoya Kinouchi, Keiko T. Kitazato, Kenji Shimada, Kenji Yagi, Yoshiteru Tada, Nobuhisa Matsushita, Yoshitaka Kurashiki, Junichiro Satomi, Masataka Sata, Shinji Nagahiro

**Affiliations:** 1 Department of Neurosurgery, Institute of Biomedical Sciences, Tokushima University Graduate School, 3-18-15, Kuramoto-cho, Tokushima, 770-8503 Japan; 2 Department of Cardiovascular Medicine, Institute of Biomedical Sciences, Tokushima University Graduate School, Tokushima, Japan

**Keywords:** Neurogenesis, Post-stroke treatment, PPARγ, Resident stem cells, Bone marrow-derived stem cells

## Abstract

Neurogenesis is essential for a good post-stroke outcome. Exogenous stem cells are currently being tested to promote neurogenesis after stroke. Elsewhere, we demonstrated that treatment with the PPARγ agonist pioglitazone (PGZ) before cerebral ischemia induction reduced brain damage and activated survival-related genes in ovariectomized (OVX) rats. Here, we tested our hypothesis that post-ischemia treatment with PGZ inhibits brain damage and contributes to neurogenesis via activated stem cells. Bone marrow (BM) cells of 7-week-old Wistar female rats were replaced with BM cells from green fluorescent protein-transgenic (GFP^+BM^) rats. Three weeks later, they were ovariectomized (OVX/GFP^+BM^ rats). We subjected 7-week-old Wistar male and 13-week-old OVX/GFP^+BM^ rats to 90-min cerebral ischemia. Male and OVX/GFP^+BM^ rats were divided into two groups, one was treated with PGZ (2.5 mg/kg/day) and the other served as the vehicle control (VC). In both male and OVX/GFP^+BM^ rats, post-ischemia treatment with PGZ reduced neurological deficits and the infarct volume. In male rats, PGZ decreased the mRNA level of IL-6 and M1-like macrophages after 24 h. In OVX/GFP^+BM^ rats, PGZ augmented the proliferation of resident stem cells in the subventricular zone (SVZ) and the recruitment of GFP^+BM^ stem cells on days 7–14. Both types of proliferated stem cells migrated from the SVZ into the peri-infarct area. There, they differentiated into mature neurons, glia, and blood vessels in association with activated Akt, MAP2, and VEGF. Post-ischemia treatment with PGZ may offer a new avenue for stroke treatment through contribution to neuroprotection and neurogenesis.

## Introduction

Brain ischemia is a leading cause of morbidity and mortality [1]. Although thrombolysis for stroke is an accepted treatment, fewer than 5% of stroke patients are treated with tissue plasminogen activator. New therapeutic strategies are needed for stroke patients.

While cerebral ischemia induces pro-inflammatory cytokines, resulting in the expansion of brain damage, it stimulates inherent neurogenesis in the subventricular zone (SVZ) and promotes the migration of newly formed neuronal progenitor cells toward the ischemic area [2]. This process may help to repair the brain and reconstruct neural networks. In rodents [3], monkeys [4], and humans [5], the SVZ, located in the lateral wall lining the lateral ventricle, harbors the largest population of neural stem cells capable of generating new neurons, astrocytes, and oligodendrocytes. While enhanced neurogenesis and angiogenesis in the SVZ and subgranular zone have been documented in adult rodent brains after focal ischemia [6], as most of the newly formed cells promptly undergo apoptosis, their effect on functional recovery is limited [2].

The administration of pioglitazone (PGZ), a peroxisome proliferator-activated receptor gamma (PPARγ) agonist, before cerebral ischemia induction protected rodents against ischemic brain damage [7, 8]. Its beneficial effects were associated with a decrease in the expression of interleukin-6 (IL-6) and caspase-3 and the improvement of neurological function in male rats [7]. However, there are few studies regarding the effects of post-ischemic treatment with PGZ.

As estrogen deficiency and menopause are thought to be major risk factors for cerebral ischemia, in an earlier study [8], we compared the effects of pre-treatment with PGZ on cerebral ischemia in oophorectomized (OVX) and non-OVX rats. We demonstrated that PGZ prevented ischemic brain damage in OVX rats when it was administered before stroke and that this was associated with the upregulation of anti-apoptotic and survival genes via the trans-activation of STAT3 and PPARγ in the peri-infarct region. This suggests its contribution to neurogenesis. However, it remained to be determined whether post-ischemic treatment with PGZ contributes to neurogenesis and neurological improvement after an ischemic insult.

The local release of PGZ promoted wound healing [9]; the regulation of monocytes/macrophages by PGZ inhibited plaque destabilization and rupture in ApoE^−/−^ mice [10], suggesting that post-stroke treatment with PGZ may be useful. Based on findings in other [7, 9, 10] and our studies [8], we hypothesized that the activation of PPARγ elicited by the administration of PGZ after stroke may be beneficial due to not only its anti-inflammatory effects but also its promotion of neurogenesis. As neurogenesis is regulated by the stem cell niche [11], we focused on this issue. To examine whether PGZ is associated with neuro- and angiogenesis, we prepared female rats whose bone marrow (BM) cells were replaced with BM cells from green fluorescent protein-transgenic (GFP^+BM^) rats before oophorectomy (OVX/GFP^+BM^ rats). We first examined the neuroprotective effects elicited by post-ischemia treatment with PGZ in male and OVX/GFP^+BM^ rats and then investigated its effects on neurogenesis in OVX/GFP^+BM^ rats.

Here, we show that even post-ischemia, early-phase treatment with PGZ inhibits the infarct size and alleviates neurological deficits in male and in OVX/GFP^+BM^ rats. We demonstrate that the decrease in the pro-inflammatory cytokine IL-6 and in M1-like macrophages associated with an increase in PPARγ after ischemia in male rats. We also document that in OVX/GFP^+BM^ rats, PGZ activated innate stem cells in the SVZ and recruitment of GFP^+BM^ stem cells with an increase in PPARγ and then increased the expression of Akt, MAP2, and VEGF in the cortical peri-infarct area, leading to neurogenesis.

## Materials and Methods

Our study was approved by the Ethics Committee of the Institute of Biomedical Sciences, Tokushima University Graduate School, and conducted in accordance with current RIGOR guidelines [12, 13] and the National Institutes of Health (NIH) Guide for the Care and Use of Laboratory Animals. We purchased male and female Wistar rats from Charles River Laboratories Japan Inc. (Yokohama, Japan). They were housed in conventional rat cages in a temperature- and humidity-controlled room (about 23 °C and 50%, respectively) under a 12-h inverted light/dark cycle and were fed standard chow. Anesthesia was with 2% isofluorane in 30% oxygen and 70% nitrous oxide. The experiments were reported according to the “Animal Research: Reporting of In Vivo Experiments (ARRIVE)” guidelines to improve the design, analysis, and reporting of research using animals—maximizing information published and minimizing unnecessary studies. All procedures were performed by investigators blinded to the treatment that rats had undergone according to a protocol approved by the Animal Care Committee of Tokushima University Medical School. For randomization, we used random-number tables; the treatment groups were identified by ear punch and the cages were labeled.

### Whole-Body Irradiation and BM Transplantation in Female Rats

The BM donors were 6-week-old GFP-transgenic rats purchased from Japan SLC, Inc. (Hamamatsu, Japan). They were killed under deep anesthesia by cervical dislocation. BM was obtained by flushing the femora and tibiae with sterile phosphate-buffered saline (PBS). BM cells were suspended in PBS, washed several times, counted, and resuspended at 4 × 10^7^ cells/ml. All procedures were as described elsewhere [14].

At the age of 7 weeks, the Wistar female rats underwent whole-body irradiation with a single 10-Gy dose. Within 24 h, thereafter, they were injected with 300 μl of the GFP-labeled BM cell suspension via the tail vein.

### Animals

Females received BM cells from GFP rats who were subjected to bilateral OVX at the age of 10 weeks (OVX/GFP^+BM^ rats). Based on our and other earlier studies [7, 8], 7-week-old male and 13-week-old OVX/GFP^+BM^ Wistar rats weighing 250–270 g and 280–300 g, respectively, were subjected to 90-min middle cerebral artery occlusion-reperfusion (MCAO-R). One group of randomly selected rats was injected intraperitoneally (i.p.) with 2.5 mg/kg/day PGZ; the other was the vehicle control (VC). In male and OVX/GFP^+BM^ rats, we recorded the effects of PGZ delivered in the early phase after MCAO-R (day 0) and once a day for 7 (males) and 14 consecutive days (OVX/GFP^+BM^ rats).

Besides examining the response to early-phase post-ischemia treatment with PGZ in OVX/GFP^+BM^ rats, we also assessed its effect on post-MCAO-R regeneration on days 7–14. The OVX/GFP^+BM^ rats were randomly sub-divided into two groups: one group received 2.5 mg/kg PGZ i.p. once a day for 14 consecutive days after MCAO-R, the other served as the VC. PGZ, a gift from Takeda Pharmaceutical Co., was dissolved in dimethylsulfoxide (DMSO) and diluted (× 3) with saline just before i.p. injection (0.4 ml/kg body weight). VC rats were injected DMSO dilution at the same concentration and volume as PGZ.

### Focal Cerebral Ischemia

During all surgical procedures, the rats were under anesthesia with 2% isofluorane in 30% oxygen and 70% nitrous oxide; their rectal temperature was monitored with a thermometer (KN-91, Natsume) and maintained at 37 ± 0.5 °C with a warming plate.

For 90-min MCAO, we inserted an intraluminal filament as described elsewhere [15, 16]. To block major collateral flow, the pterygopalatine artery was ligated at its origin. The internal and common carotid artery were transiently occluded with loosely tied 3–0 silk sutures; a silicon-coated 4–0 nylon thread was introduced into the external carotid artery and advanced into the internal carotid artery to occlude the proximal orifice of the MCA. To confirm MCAO, we used a laser-Doppler flow probe (Unique Medical, Osaka, Japan) to measure the blood flow at the temporal bone surface at a site 1 mm posterior to the bregma and 3 mm inferior to the temporal line. MCAO reduced the blood flow to 20–30% of the baseline.

Rats with successful MCAO-R consistently exhibited circling behavior, decreased resistance to lateral push, forelimb flexion, and shoulder adduction. We excluded around 10% of the rats because MCAO-R was incomplete. Blood glucose levels were determined in whole venous blood with an automatic glucose meter (Accu-check Aviva blood glucose meter, Roche Diagnostics, Tokyo, Japan). The blood pressure was measured by telemetry (Data Science Inc., MN55126, USA) before, during, and after MCAO-R and recorded using the Dataquest Advanced Research Technologies Acquisition program (Unique Medical).

### Measurement of the Infarct Volume

The brains were extracted and equal 2-mm-spaced slices and six coronal blocks were prepared immediately using a brain matrix (Bioresearch Center, Nagoya, Japan). The samples did not contain olfactory tissue or tissue from the cerebellum. All but the 3rd coronal sections were immersed in a 2,3,5-triphenyltetrazolium chloride (TTC) solution in PBS to detect the infarct area. We identified the area surrounding the infarct area in the frontal cortex as the peri-infarct area. The tissue samples were stored at − 80 °C until Western blot analysis and determination of the mRNA level. The extent of ischemic infarction was traced manually, and the integrated volume was calculated using NIH 1.36b Image J software. Artifacts from brain edema were eliminated by applying the indirect measurement method based on the contralateral brain volume.

### Neurological Assessment

Neurological deficits were assessed by an examiner blinded to the treatment the rats had undergone. We modified the neurological scoring system of Huang et al. [17] and Chen et al. [18] and recorded our findings as 0 = normal; 1 = forelimb or hindlimb flexion, head turned > 10 to the vertical axis within 30 s after raising the rat by the tail, inability to walk straight on the floor, and grasping the side of the beam during the beam balance test; 2 = circling toward the paretic side, one limb falling off the beam; 3 = falling to the paretic side, hugging the beam, and two limbs falling off the beam; 4 = attempting to balance on the beam but falling off (> 40 s); 5 = attempting to keep balance on the beam but falling off (> 20 s); and 6 = falling off the beam without attempting to balance or hang on to the beam (< 20 s). The rats were evaluated immediately after successful MCAO, 24 h after MCAO-R, and again on days 1, 3, and 7 (males) and on days 1, 3, 7, and 14 (OVX/GFP^+BM^ rats) after treatment with PGZ or VC. The total maximum score was 12.

### Quantitative Real-Time PCR

Total RNA obtained from the peri-infarct area was isolated with the BioRobot EZ1 and EZ1 universal tissue kit (Qiagen, Tokyo, Japan). RNA was converted to cDNA using the transcript first-strand cDNA synthesis kit (Qiagen). Quantitative real-time PCR assay of each sample was on Light Cycler FastStart DNA Master SYBR Green I and Roche LightCycler 2.0 (Roche Diagnostics, Tokyo, Japan) instruments. Primers for GAPDH were from Roche and used according to the manufacturer’s directions. The other primers were: for rat IL-6, forward (F), 5′-TCT CAG GGA GAT CTT GGA AAT G-3′, reverse (R), 5′-TAG AAA CGG AAC TCC AGA AGA C-3′; for rat TNF-α, (F), 5′-CCC AAC AAG GAG GAG AAG T-3′, (R), 5′-CGC TTG GTG GTT TGC TAC-3′; for rat IL-1β, (F), 5′-TGC AGG CTT CGA GAT GAA C-3′ (R), 5′-AGC TCA TGG AGA ATA CCA CTT G-3′; for rat VEGF, (F), 5′-CACATAGGAGAGATGAGCTT-3′, (R), 5′-CTGGCTTTG TTCTATCTTTC-3′. The amplified product was separated on 1.5% agarose gels containing EtBr solution (Wako, Osaka, Japan) and visualized on an ultraviolet transilluminator. The results were normalized to the expression of GAPDH mRNA.

### Immunohistochemistry

The rats were transcardially perfused with 4% paraformaldehyde in PBS on ice. Their brains were fixed and 6-μm-thick frozen sections were mounted on Matsunami adhesive silane (MAS)-coated glass slides (Matsunami Glass, Tokyo, Japan), blocked with serum-free protein (DakoCytomation), and then the slides were incubated with primary antibodies diluted with Canget signal immunostain (Toyobo, Osaka, Japan). The antibodies were rabbit polyclonal antibody against PPARγ (Abcam, Tokyo, Japan), GFAP, Nestin, MAP2, (Santa Cruz Biotechnology), and antibodies against GFP (Cell Signaling Technology). We used mouse monoclonal antibody against neuronal nucleus (NeuN) (Millipore, Tokyo, Japan), Musashi-1 (AbD Serotec), CD16, CD68 (Santa Cruz Biotechnology), CD31 (MAB1393, CHEMICON, MA), caspase-3, GFP, and Nestin (Cell Signaling Technology). The tissue samples were mounted with Vectashield (Vector Laboratories Inc., Burlingame, CA). Visualization was with Alexa Fluor 594 donkey anti-rabbit IgG or 488 goat anti-mouse IgG (Molecular Probes, Eugene, OR); the slides were examined under a fluorescence microscope (KEYENCE, BZ-X710, Osaka, Japan). To examine the specificity of immunoreactivity, the primary antibody was omitted to provide a nonspecific control. A parallel set of tissue sections was subjected to hematoxylin and eosin staining to identify the infarct core and the peri-infarct region. We counted all cells positive for Musashi-1 and GFP in the peri-infarct area. Images were captured at ×10 magnification under the microscope. In each animal, we randomly selected two areas containing positive cells in 150 × 150-μm fields around the peri-infarct area. Tissue samples from four rats in each group were analyzed by densitometry of the positive cells using BZH-3A (KEYENCE) analysis software.

### Bromodeoxyuridine (BrdU) Labeling in OVX/GFP^+BM^ Rats

We recorded the time course of proliferating cells in the brain after cerebral ischemia by pulse-labeling. BrdU, a thymidine analogue that is incorporated into the DNA of dividing cells during the S-phase, was used for mitotic labeling (Sigma Chemical) [19]. We applied a cumulative labeling method to examine the population of proliferating cells in OVX rats exposed for 14 days to cerebral ischemia and injected BrdU (50 mg/kg, i.p.) every 4 h for 12 h before killing the rats on the 1st, 3rd, 7th, or 14th day after the induction of cerebral ischemia (*n* = 6 each). BrdU-positive cells were detected immunohistochemically using sheep polyclonal antibody BrdU (LifeSpan BioSciences Inc.) as the primary and donkey anti-sheep IgG (Molecular Probes, Eugene, OR) as the secondary antibody.

### Western Blot Analysis in OVX/GFP^+BM^ Rats

Brain tissue in the peri-infarct area was homogenized and sonicated in RIPA buffer (Thermo Scientific, Rockford, IL) containing phosphatase and protease inhibitors (Roche, Tokyo, Japan) and centrifuged. Total protein in the supernatant was measured with the BCA protein assay kit (Pierce, Rockford, IL). Protein was separated by 7.5 or 12% SDS-PAGE and transferred to a polyvinylidenedifluoride membrane. After blocking with 5% skim milk or BSA in Tris-buffered saline solution-Tween 20 (T-TBS), the membrane was incubated with the primary antibodies in Canget signal immunostain or T-TBS. The same primary antibodies as used for the immunohistochemical studies, rabbit polyclonal antibody against p-Akt (Cell Signaling Technology), MAP2, and mouse monoclonal anti-β-actin (Sigma, Tokyo Japan) were used. After incubation with horseradish peroxidase-conjugated secondary antibodies (GE Healthcare, Buckinghamshire, UK), signals were detected by chemiluminescence using an ECL-plus kit (GE Healthcare). Images were analyzed with Image Quant LAS 4000 mini (GE Healthcare) and Image J software and quantified as the relative increase over the controls after normalization with β-actin.

### Statistical Analysis

Power estimates were calculated based on *α* = 0.05, 1-β = 0.8, and a surgery-related drop-out rate of around 10% to obtain group sizes appropriate for detecting an effect size of 0.4 based on a preliminary experiment using G*Power 3.1. The infarct volume and the neurological score were analyzed with Student’s *t* test and Man-Whitney *U* test, respectively; their correlation was assessed by the Spearman’s rank-correlation coefficient. The mRNA expression levels were determined with analysis of variance (ANOVA) followed by Scheffe’s test for three-group comparisons. Statistical analyses were performed using IBM SPSS Statistics 22. Data are shown as the mean ± SD. Differences were considered statistically significant at *p* < 0.05.

## Results

### Male Rats: Treatment with PGZ in the Early Post-ischemia Phase Reduced the Cerebral Infarct Size and Ameliorated Neurological Deficits by Inhibiting Pro-inflammatory Responses

**Fig. 1:**
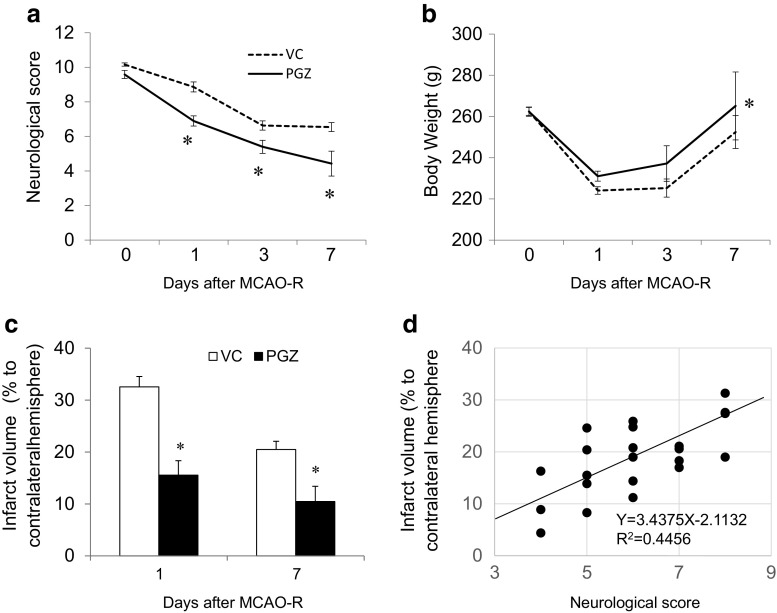
Effects of PGZ against brain damage in male rats. Post-ischemia treatment with PGZ or vehicle was performed immediately after MCAO induction on day 0 and once a day for 7 consecutive days. The neurological scores (**a**), body weight (**b**), and infarct volume (**c**) were recorded in PGZ- and vehicle-treated male rats. The infarct volume was recorded as a percentage of the contralateral hemisphere using Image J software (each group *n* = 12). The correlation between the infarct volume and neurological deficits was assessed by Spearman’s rank-correlation coefficient (**d**). Each bar represents the mean ± SD. *p < 0.05 vs. VC by ANOVA followed by Scheffe’s test. MCAO-R middle cerebral artery occlusion-reperfusion, VC vehicle control, PGZ 2.5 mg/kg pioglitazone

**Fig. 2:**
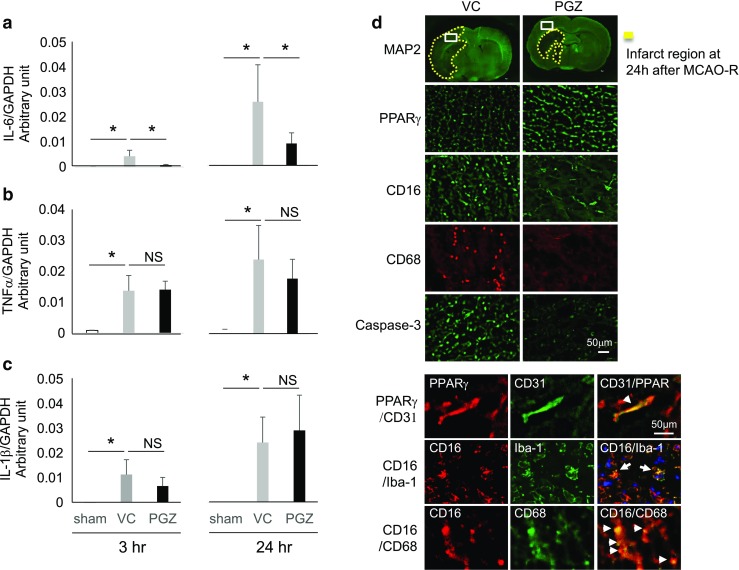
The mRNA level of pro-inflammatory cytokines and representative immunohistochemistry findings in PGZ- and VC-treated male rats. The mRNA level of the pro-inflammatory cytokines IL-6 (**a**), TNFα (**b**), and IL-1β (**c**) was assessed by quantitative real-time PCR assay and normalized by GAPDH. Data obtained 3 and 24 h after MCAO-R are shown. Data are the mean ± SD from eight rats per group. *p < 0.05 vs. VC by ANOVA followed by Scheffe’s test. The expression of PPARγ, CD16, CD68, and caspase-3 was examined in the peri-infarct region of PGZ-treated and VC rats 24 h after MCAO (**d**). PPARγ- and CD16-positive cells were co-localized with VEGF-, Iba-1- or CD68-positive cells

We first assessed the effects of PGZ treatment in the early phase after experimental cerebral ischemia. Compared to the VC males, on days 1–7 after the ischemic insult, PGZ rats manifested a lower neurological score and earlier recovery of the body weight loss (Fig. 1a, b). The infarct volume was smaller than in VC rats (Fig. 1c) and correlated with the neurological score (Fig. 1d). Next, to address the mechanisms underlying the effects of PGZ, we examined its anti-inflammatory effects against brain ischemic injury. In VC rats, the mRNA level of IL-6, IL-β, and TNFα was increased 3 h after ischemia induction and augmented at 24 h (Fig. 2a–c). In PGZ rats, the mRNA level of IL-6 (Fig. 2a) but not of IL-β and TNFα was significantly decreased at 3 and 24 h (Fig. 2b, c). Immunohistochemically, the expression of PPARγ was higher in PGZ than VC rats; CD16- and CD68-positive cells were fewer and the expression of caspase-3 was lower (Fig. 2d). PPARγ was localized in CD31-positive cells. CD16-positive cells were Iba-1 or CD68 positive. Treatment with PGZ in the early phase post-ischemia appeared to exert beneficial effects through anti-apoptosis and anti-inflammatory response effects elicited by the expression of PPARγ.

### OVX/GFP^+BM^ Rats: Increased PPARγ Expression Elicited by PGZ Was Associated with Neuroprotection and the Activation of Resident Stem Cells in the SVZ and the Recruitment of BM Cells

**Fig. 3:**
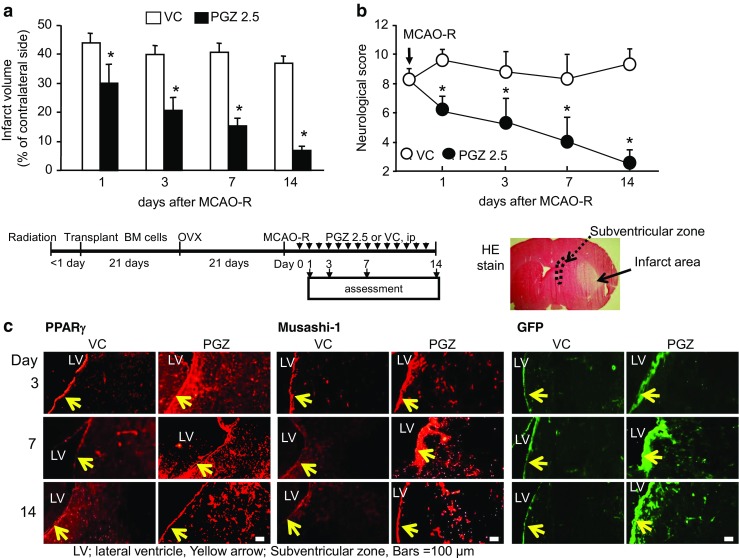
Effects of post-ischemia treatment with PGZ in OVX/GFP^+BM^ rats. PGZ or vehicle (VC) was administered immediately after MCAO induction on day 0 and once a day for 14 consecutive days. The infarct volume (**a**) was recorded in OVX/GFP^+BM^ rats treated with PGZ or VC (each group *n* = 12). The neurological score (**b**) was assessed as described in Materials and Methods. Each bar represents the mean ± SD *p < 0.05 vs. VC by ANOVA followed by Scheffe’s test. Representative immunohistochemistry findings for PPARγ, Musashi-1, and GFP (**d**) in the SVZ after MCAO-R

Our earlier study in ovariectomized (OVX) rats treated with PGZ before ischemia induction suggested neurogenesis in the post-ischemic phase [8]. To examine the role in neuroprotection and neurogenesis of early-phase PGZ treatment after ischemia induction, we used OVX/GFP^+BM^ rats whose BM cells were replaced by BM cells from GFP rats. While 75% of the VC-OVX/GFP^+BM^ rats died within 14 days after MCAO-R, all OVX/GFP^+BM^ rats treated with PGZ (PGZ rats) survived (*n* = 12 in each group). On days 1, 3, 7, and 14 after MCAO-R, the cerebral infarct volume was significantly smaller in PGZ than VC rats (*p* < 0.05, Fig. 3a) and their neurological deficits were significantly less pronounced (*p* < 0.05, Fig. 3b), indicating neuroprotection by early-phase PGZ treatment. There was no significant difference in the cerebral blood flow, blood glucose levels, and blood pressure between the PGZ and VC rats during and after MCAO-R (data not shown).

Compared to the contralateral non-ischemic side of VC rats, on days 3 and 7 post-MCAO-R, the expression of PPARγ and of Musashi-1- and GFP-positive cells was increased in the SVZ on the infarct side of PGZ rats (Fig. 3c). The expression of PPARγ in the SVZ seemed to be associated with an increase in innate stem cells and the recruitment of allogeneic BM-derived stem cells.

### OVX/GFP^+BM^ Rats: PPARγ Activation Promoted the Proliferation of Stem Cells in the SVZ and their Translocation to the Peri-infarct Region

**Fig. 4:**
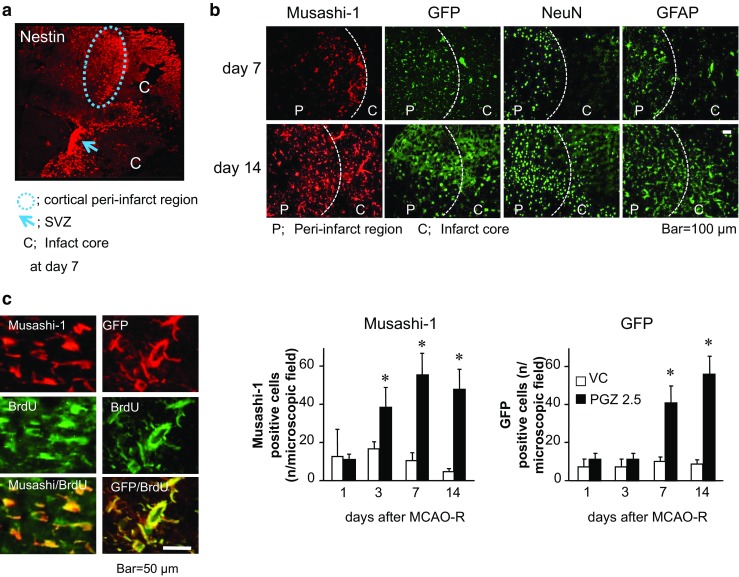
Stem cells migration from the SVZ into the cortical peri-infarct region promoted by PGZ in OVX/GFP^+BM^ rats. Representative nestin-positive cells in the SVZ and the cortical peri-infarct region on day 7 after ischemia induction in PGZ-treated OVX/GFP^+BM^ rats (**a**). Cells positive for Musashi-1, GFP, NeuN, and GFAP were detected in the infarct- and the peri-infarct area (**b**). Musashi-1- and GFP-positive cells were counted in 150 × 150-μm fields. The total cell number (*n*) in each area was 140–160 cells for DAPI. Each bar represents the mean ± SD from two areas in each of four rats. *p < 0.05 vs. VC by ANOVA followed by Scheffe’s test. Representative Musashi-1- and GFP-positive cells incorporated BrdU (**c**)

As shown in Fig. 4a, on the 7th day after ischemia induction, nestin-positive cells were detected in the SVZ and the cortical peri-infarct area of PGZ rats; the cells seemed to migrate from the SVZ to the peri-infarct region. The number of translocated Musashi-1-positive- and of GFP-positive cells was significantly higher in PGZ than VC rats (*p* < 0.05, Fig. 4b) and associated with the increase in NeuN- and GFAP-positive cells. These cell populations seemed to include both resident stem cells in SVZ and recruitment of BM-derived stem cells replaced by allogeneic BM cells. We immunohistochemically assessed the incorporation of the cell proliferation marker BrdU in the stem cells. The presence of many BrdU-labeled Musashi-1- and GFP-positive cells was evidence for the proliferation of stem cells in the peri-infarct region (Fig. 4c). PPARγ activation may promote not only the proliferation of stem cells in the SVZ but also the migration of proliferated stem cells from the SVZ to the cortical peri-infarct region.

### OVX/GFP^+BM^ Rats: Proliferated Neuronal Stem Cells Differentiated into Blood Vessels, Neurons, and Glia in the Cortical Peri-infarct Region Via the Upregulation by PGZ of the Survival Signal Pathway

**Fig. 5:**
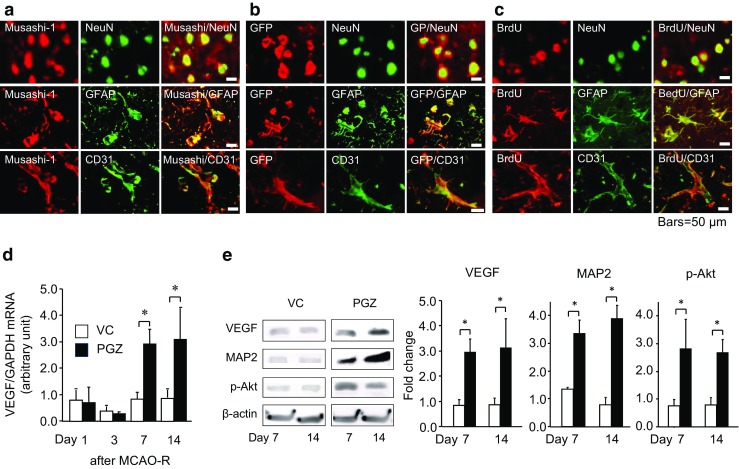
Stem cells differentiation into blood vessels, neurons, and glia via the upregulation of the survival signal pathway induced by PGZ in OVX/GFP^+BM^ rats. In the peri-infarct region, double immunohistochemical staining for Musashi-1 (**a**), GFP (**b**), and BrdU (**c**) shows co-localization with NeuN, GFAP, and CD31, respectively. The mRNA level of VEGF (**d**) was analyzed by quantitative PCR. The protein expression of VEGF, p-Akt, and MAP2 in the peri-infarct region (**e**) was detected on Western blots and analyzed using LAS 4000 and Image J software. Data are the mean ± SD from eight rats per group. *p < 0.05 vs. VC by ANOVA followed by Scheffe’s test

**Fig. 6:**
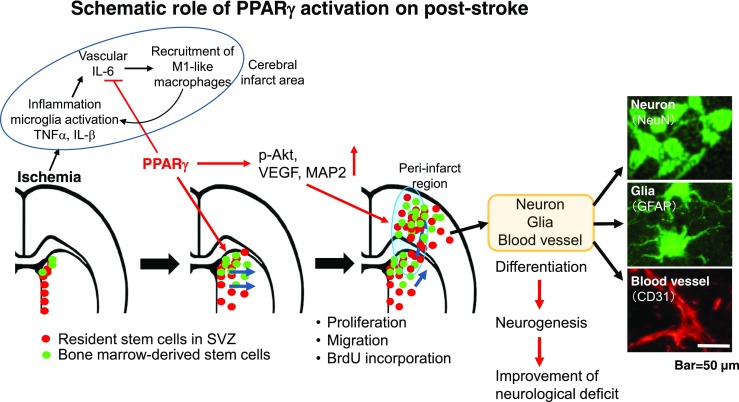
Schematic role of PGZ administered in the early phase after cerebral ischemia. Activation of PPARγ by PGZ inhibits pro-inflammatory responses and promotes neurogenesis in the peri-infarct region. Some NeuN (green)-, GFAP (green)-, or CD31 (red)-positive cells were mature neurons, extended axons, glias, and blood vessels

Lastly, we examined the effects of PPARγ activation by PGZ on neurogenesis. Some of the Musashi-1- and GFP-positive cells were NeuN, GFAP, or CD-31 positive (Fig. 5a, b). BrdU-labeled cells were also NeuN, GFAP, or CD-31 positive and some of them were mature, and seen in neurons, glia, and blood vessels. Extended axons were also observed (Figs. 5a–c, 6), suggesting that PGZ promoted the differentiation into mature neurons, glia, and blood vessels in the cortical peri-infarct region.

Examination of the gene and protein expression of survival- and proliferation-related molecules elicited by treatment with PGZ showed that on days 7 and 14, the mRNA level of VEGF was increased in the peri-infarct region (*p* < 0.05, Fig. 5d). Consequently, the expression of VEGF, MAP2, and p-Akt was significantly higher in PGZ than VC rats (*p* < 0.05, Fig. 5e). These findings suggest that the upregulation by PGZ of survival signaling pathways was associated with the proliferation of neuronal stem cells and their differentiation into mature neurons, glia, and blood vessels.

## Discussion

We first demonstrate that post-ischemia treatment with PGZ in early phase prevented the expansion of brain injury and attenuated neurological deficits in male and OVX/GFP^+BM^ rats. We showed that the neuroprotective effects of PGZ in the early post-ischemia phase were associated with an anti-inflammatory response involving a decrease in IL-6 and M1-type macrophages in male rats. Furthermore, we found that in OVX/GFP^+BM^ rats PGZ promoted the proliferation of both innate stem cells in the SVZ and BM-derived stem cells and their translocation from the SVZ into the cortical peri-infarct region on days 7–14 of post-ischemia. This was associated with the high expression of VEGF, MAP2, and p-AKT and an increase in PPARγ in the peri-infarct area and the differentiation of stem cells into mature cells, resulting in the alleviation of neurological dysfunction. Our findings suggest that early-phase treatment with PGZ, even after experimentally induced cerebral ischemia, contributed to a good stroke outcome in rats via its anti-inflammatory effects and the elicitation of neurogenesis (Fig. 6).

Lambertsen et al. [20] who studied experimental and human stroke, reported that within 1–6 h post-ischemia, the protein level of IL-1β and TNFα but not of IL-6 was increased in the brain and cerebrospinal fluid and persisted at high levels until 24 h. In our animal study, the mRNA level of IL-6, TNFα, and IL-1β was increased 24 h after ischemia induction; this was associated with the presence of M1-like macrophages and the expansion of cerebral infarction although the mRNA level of IL-6 was low during the first 3 h. PGZ suppressed the expression of IL-6 at 24 h without affecting TNFα and IL-1β. During the early post-ischemic phase in male rats, the different effects of PGZ against these molecules may be associated with their different expression profile after ischemia and the timing of PGZ treatment. Nakashiro [10] reported that PGZ decreased the expression of circulating inflammatory monocytes. As PGZ inhibited the expression of CD16^+^/CD68^+^ M1-like macrophages in our rats, we think that in the early phase of post-ischemia, it inhibited the recruitment of M1-like monocyte-derived macrophages from the perivascular area into the brain. According to Patzer et al. [21], IL-6-expressing microglia/macrophages in the brain were activated in the initial post-ischemic stage and immunohistochemical and Western blot analysis showed that PGZ reduced the expression of IL-6. Thus, the down-regulation of IL-6 in the early post-ischemic phase may be crucial for a reduction in ischemic brain damage.

Although we do not know how PGZ increases the expression of PPARγ in areas adjacent to the SVZ, elsewhere [8], we demonstrated that treatment with PGZ before ischemia induction upregulated STAT3 and increased the expression of anti-apoptotic bcl-2 and VEGF. IL-6 is associated with the JAK/STAT pathway and elicits a pro-inflammatory response in the early phase; in the late phase, it is associated with neurotrophic effects [22]. The regulation of IL-6 by PGZ may exert ambivalent beneficial effects in the early and late phase after stroke.

Neuronal stem cells are primordial, multipotent, self-renewing cells that give rise to differentiated progeny within all neuronal and glial lineages. They continue to produce new neurons throughout life in the SVZ and the dentate gyrus of the hippocampus [19, 23]. However, most of the newly formed cells promptly undergo apoptosis, possibly due to unfavorable conditions after cerebral ischemia and the lack of adequate trophic support [6]. In contrast, PGZ-induced stem cells seem to be less affected by ischemic conditions. The increased expression of MAP2 and p-Akt elicited by PGZ and the upregulation of anti-apoptotic genes [8] might contribute to the activation of survival signaling pathways to nourish the stem cells. Thus, the activation of PPARγ by PGZ after the ischemic insult may not only accelerate the proliferation of resident stem cells in the SVZ and the recruitment of BM-derived stem cells but may also contribute to their differentiation in the peri-infarct area.

Neurotrophic factors regulate the survival, proliferation, and differentiation of cells in the central nervous system [6, 23]. VEGF, identified as an angiogenic and vascular permeability factor, is recognized as a neurotrophic factor [24] whose role depends on its temporal and spatial profiles. During the acute phase of ischemic injury, the upregulation of VEGF in cerebral vessels increases the permeability of the blood-brain-barrier, thereby exacerbating ischemic cell damage [25]. At a later stage after stroke, it triggers angiogenesis, promotes the blood supply to the brain, and accelerates neurogenesis [26]. Despite the high expression of PPARγ elicited by PGZ in both the early and late phase after the ischemic insult imposed on our rats, the VEGF expression pattern was different in these phases. The upregulation of VEGF on days 7 and 14 may have contributed to angiogenesis. The phosphatidylinositol-3 kinase (PI3)/Akt signaling pathway plays a central role in regulating the growth, proliferation, and survival of cells under physiological and pathophysiological conditions. Its activation protects vascular function [27], promotes cell survival, and suppresses apoptosis. As did others [28, 29], we found that treatment with PGZ even after an ischemic insult increased the expression of p-Akt and MAP2 in the peri-infarct region. Therefore, elicitation of angiogenesis and neurogenesis may be attributable to the upregulation of survival signaling pathways by PGZ.

Although attempts to promote neurogenesis by injecting exogenous stem cells have remained unsuccessful, the combined delivery of PGZ and exogenous stem cells may help to promote neurogenesis. We need further studies to determine whether PGZ helps to promote the survival and proliferation of exogenous stem cells. Kernan et al. [30] reported that 4.8-year PGZ treatment of patients with insulin resistance, ischemic stroke, and transient ischemic attacks reduced the risk for recurrent stroke or myocardial infarcts but increased the risk for weight gain, edema, and bone fracture. Therefore, the condition of patients treated with PGZ must be monitored carefully, and its administration should be short term.

In conclusion, we provide new evidence that PGZ treatment in the early phase after an experimentally induced ischemic insult ameliorated neurological dysfunction and suppressed the infarct size in male and OVX/GFP^+BM^ rats. We show that its beneficial effects were associated with the induction of anti-inflammatory responses in the early and with the elicitation of angiogenesis and neurogenesis in the late post-ischemic phase. Treatment with PGZ in the early post-ischemic phase may help to limit ischemic brain damage and to alleviate neuronal deficits. We are continuing to assess the potential role of PGZ in the activation of post-stroke PPARγ because such findings may lead to treatments that improve the outcomes in stroke patients.

### Funding

This work was supported by a Grant-in-Aid for Scientific Research [JSPS KAKENHI Grant Number JP15K10306] and a Grant-in-Aid for the Strategic Young Researcher Overseas Visits Program for Accelerating Brain Circulation from the Japan Society for the Promotion of Science [JSPS Grant Number JPS2407].
